# Expression of Tim‐1 in primary CNS lymphoma

**DOI:** 10.1002/cam4.930

**Published:** 2016-10-05

**Authors:** Wataru Kishimoto, Momoko Nishikori, Hiroshi Arima, Hiroaki Miyoshi, Yuya Sasaki, Toshio Kitawaki, Kotaro Shirakawa, Takeharu Kato, Yoshitaka Imaizumi, Takayuki Ishikawa, Hitoshi Ohno, Hironori Haga, Koichi Ohshima, Akifumi Takaori‐Kondo

**Affiliations:** ^1^ Department of Hematology and OncologyGraduate School of MedicineKyoto UniversityKyotoJapan; ^2^ Department of PathologyKurume University School of MedicineAsahimachiKurumeFukuokaJapan; ^3^ Department of HematologyNagasaki University HospitalSakamotoNagasakiJapan; ^4^ Department of HematologyKobe City Medical Center General HospitalKobeHyogoJapan; ^5^ Department of HematologyTenri HospitalMishima‐choTenriNaraJapan; ^6^ Department of PathologyKyoto University HospitalKyotoJapan

**Keywords:** Biomarker, central nervous system, diffuse large B‐cell lymphoma, immunohistochemistry, Tim‐1

## Abstract

Primary central nervous system lymphoma (PCNSL) is a distinct subtype of extranodal lymphoma with aggressive clinical course and poor outcome. As increased IL‐10/IL‐6 ratio is recognized in the cerebrospinal fluid (CSF) of PCNSL patients, we hypothesized that PCNSL might originate from a population of B cells with high IL‐10‐producing capacity, an equivalent of “regulatory B cells” in mice. We intended in this study to clarify whether Tim‐1, a molecule known as a marker for regulatory B cells in mice, is expressed in PCNSL. By immunohistochemical analysis, Tim‐1 was shown to be positive in as high as 54.2% of PCNSL (26 of 58 samples), while it was positive in 19.1% of systemic diffuse large B‐cell lymphoma (DLBCL) samples (17 of 89 samples; *P *<* *0.001). Tim‐1 expression positively correlated with IL‐10 expression in PCNSL (Cramer's *V* = 0.55, *P *<* *0.001), and forced expression of Tim‐1 in a PCNSL cell line resulted in increased IL‐10 secretion, suggesting that Tim‐1 is functionally linked with IL‐10 production in PCNSL. Moreover, soluble Tim‐1 was detectable in the CSF of PCNSL patients, and was suggested to parallel disease activity. In summary, PCNSL is characterized by frequent Tim‐1 expression, and its soluble form in CSF may become a useful biomarker for PCNSL.

## Introduction

Primary central nervous system lymphoma (PCNSL) is an uncommon form of extranodal non‐Hodgkin's lymphoma that arises within the central nervous system (CNS), most cases of which demonstrate diffuse large B‐cell lymphoma (DLBCL) histology 1. Although the etiology for the development of lymphoma in this extranodal site remains unclear, PCNSL patients are known to be characterized by high IL‐10/IL‐6 ratio in the cerebrospinal fluid (CSF), and it may be speculated that PCNSL is derived from B cells with IL‐10‐producing capacity.

In mouse models of multiple sclerosis (experimental autoimmune encephalomyelitis, EAE) and cerebral infarction, a population of IL‐10‐producing B cells, named regulatory B cells, has been shown to have a function to migrate to the CNS and suppress inflammation 2, 3, 4. These regulatory B cells are reported to express a cell surface molecule T‐cell immunoglobulin domain and mucin domain protein 1 (Tim‐1) at a high frequency 5.

Tim‐1 is a type I cell surface glycoprotein that is expressed on various immune cells, such as activated T cells, B cells, natural killer T (NKT) cells, and dendritic cells, and regulates diverse immune responses 6, 7, 8, 9, 10, 11, 12. It is also known to be induced on tubular epithelial cells following kidney injury 13, and it is then cleaved at a membrane proximal point and the extracellular domain of the protein is released into the urine 14, 15, 16. In B cells, Tim‐1 is shown to be expressed by a large majority of IL‐10‐expressing population 5. Regulatory B cells with defective Tim‐1 mutation have a profound defect in IL‐10 production, suggesting that Tim‐1 plays a pivotal role in their IL‐10 production 17.

We hypothesized that PCNSL may originate from such B cells that have a physiological role in producing IL‐10 to suppress unfavorable inflammation in the CNS. In this study, we found that Tim‐1 was detected in more than half of the PCNSL samples by immunohistochemistry, and Tim‐1 was also detectable in soluble form in the CSF of PCNSL patients with active disease. Our findings suggest that PCNSL is characterized by frequent Tim‐1 expression, and detection of soluble Tim‐1 in CSF may be useful for the diagnosis and evaluating the disease activity of PCNSL.

## Materials and Methods

### Clinical samples, immunohistochemistry, and ELISA

For immunohistochemical analysis, formalin‐fixed, paraffin‐embedded samples of 48 PCNSL (median age 63; age range 16–86 years) and 89 systemic DLBCL patients (median age 67; age range 22–86 years) diagnosed at Kyoto University Hospital, Nagasaki University Hospital, Kobe Central Citizen Hospital, and Tenri Hospital were used. All PCNSL samples were obtained from immunocompetent patients at first diagnosis, and were of DLBCL histology. They were stained with anti‐Tim‐1 antibody (MAB 1750; R&D Systems, Minneapolis, MN), anti‐IL‐10 antibody (AF‐217; R&D Systems), anti‐CD3 antibody (2GV6; Ventana Medical Systems, Tucson, AZ), and anti‐CD20 antibody (L26; DAKO, Carpinteria, CA). Cut‐off values of Tim‐1 and IL‐10 positivity were set as 30%, according to the criteria that are commonly applied to the IHC analysis of lymphoma 18. Statistical analysis was performed using STATA 11 software (Stata Corporation, College Station, TX). The Chi‐square test, Mann–Whitney U‐test, t‐test, and paired t‐test were used for comparison between two groups. Fluorescent double staining was performed by labeling anti‐Tim‐1 antibody with FITC‐conjugated donkey anti‐mouse antibody (ab98770; Abcam, Cambridge, MA), and anti‐IL‐10 antibody with rabbit anti‐goat antibody (E0466; DAKO, Carpinteria, CA), and Alexa555‐conjugated donkey anti‐rabbit antibody (ab150070; Abcam). Nuclei were counterstained with DAPI (H‐1200; Vector Laboratories; Burlingame, CA).

CSF samples were collected from lymphoma patients after obtaining written informed consent, and were centrifuged at 2000*g* for 10 min at 4°C and stored at −80°C until analysis. Soluble Tim‐1 levels of the CSF samples were analyzed by ELISA using Human Tim‐1/KIM‐1/HAVCR Duoset (R&D Systems) following the manufacturer's instructions. These clinical samples were obtained and used under the approval of the Institutional Review Board of each institute, and performed in accordance with the ethical standards of an institutional research committee and the provisions of the Declaration of Helsinki (as revised in 2013).

### Gene expression analysis

The expression levels of *TIM‐1* and *IL‐10* genes were compared using the previously published datasets available in the NCBI GEO database (http://www.ncbi.nlm.nih.gov/geo/). We picked up GEO series data and obtained the cell intensity files from the database. The CEL files were imported into the R software package (ver. 3.1.1., Free Software Foundation, Boston, MA), and the probe‐level data were converted into normalized expression profiles using the Affy package 19. The expression levels of each gene were normalized using *ACTB*.

### Cell lines and immunoblot analysis

A PCNSL cell line, TK (JCRB1206), was obtained from JCRB Cell Bank (Tokyo, Japan). A Burkitt lymphoma cell line, Raji, a mantle cell lymphoma cell line, Granta 519, and a mouse fibroblast cell line, L cells, were as described elsewhere 20, 21, 22. These cell lines were cultured in RPMI‐1640 medium supplemented with 10% (or 20% for TK cells) heat‐inactivated fetal calf serum (FCS), 100 *μ*g/mL penicillin, 100 *μ*g/mL streptomycin, and 2 mmol/L L‐glutamine. A human embryonic kidney cell line, 293T, was cultured in DMEM containing 10% FCS, penicillin, streptomycin, and L‐glutamine.

For immunoblot analysis, the cells were lysed in lysis buffer (25 mmol/L HEPES [pH 7.4], 150 mmol/L NaCl, 1 mmol/L MgCl_2_, 1 mmol/L EDTA, 1.0% Triton X‐100, 10% glycerol, and protease inhibitor cocktail). Total cell lysates and cell supernatants of the culture medium were subjected to sodium dodecyl sulfate–polyacrylamide gel electrophoresis and transferred to Immobilon‐P polyvinylidene difluoride transfer membranes (Millipore, Bedford, MA). The membranes were immunoblotted with anti‐Tim‐1 antibody (clone 219211; R&D Systems) or anti‐FLAG M2 monoclonal antibody (Sigma, St. Louis, MO). The membranes were subsequently incubated with horseradish peroxidase‐conjugated anti‐mouse secondary antibody, and immunoreactive proteins were visualized by enhanced chemiluminescence reaction using SuperSignal West Pico Chemiluminescent Substrate (Thermo Scientific, Rockford, IL).

### TIM‐1 transfection assay

Human *TIM‐1* transcript was PCR amplified from cDNA of the lung adenocarcinoma cell line A549, which is known to express a high level of Tim‐1 23, and subcloned into pFLAG‐CMV‐5a expression vector (Sigma). The vector was transduced into 293T cells using X‐tremeGENE HP DNA Transfection Reagent (Roche, Mannheim, Germany). Flag‐tagged Tim‐1 was also subcloned into a pCS‐CAG‐EGFP lentiviral vector, which was constructed by replacing the CD19 promoter of E*μ*mar‐L.CD19‐EGFP vector with CAG promoter 24, 25. The packaging plasmid pCAG‐HIVgp and the VSV‐G‐ and Rev‐expressing plasmids (pCMV‐VSV‐G‐RSV‐Rev) were kindly provided by Dr. H. Miyoshi, RIKEN Bioresource Center, Tsukuba, Japan. *TIM‐1* expression vector was transfected into 293T cells with packaging plasmids, and viral supernatants were collected after 48 h, concentrated by ultracentrifugation at 20,000*g* for 2 h, and transduced in TK cells.

*TIM‐1* or mock‐introduced 293T and TK cells were incubated for 12 h in a serum‐free medium, and the cells and supernatants were separately collected and analyzed for Tim‐1 protein expression by immunoblotting. For cell viability assay, TK cells were resuspended in RPMI‐1640 medium with 20% FCS at a concentration of 4 × 10^6^/mL and seeded in a 96‐well plate. After 24 and 48 h of culture, IL‐10 production from TK cells introduced with mock or TIM‐1 expression vectors was analyzed by ELISA, using Human IL‐10 Duoset (R&D Systems) following the manufacturer's instructions. The cells were cultured with 15 *μ*g/mL cisplatin or 20 *μ*mol/L dexamethasone for 12 and 24 h, and cell apoptosis was evaluated by propidium iodide (PI) staining (Biolegend, San Diego, CA) and FACS analysis.

### T‐cell proliferation and cytokine production analysis

*TIM‐1*‐expressing or mock vector was transfected into L cells, and supernatants were collected after culturing for 12 h. Peripheral blood mononuclear cells of three healthy donors were first separated from peripheral blood on a Ficoll‐paque density gradient (Sigma), and subsequently CD4^+^ and CD8^+^ T cells were collected using CD4 and CD8 MicroBeads and a magnetic‐activated cell sorting (MACS) system (Miltenyi Biotec, Bergisch‐Gladbach, Germany). CD4^+^ and CD8^+^ T cells were individually resuspended in the supernatants of L cells at a concentration of 1 × 10^6^/mL, seeded in a 96‐well plate, and cultured under stimulation with anti‐CD3/CD28 beads (Life Technologies, Gaithersburg, MD). After 48 or 72 h of culture, cell numbers were counted and their supernatants were analyzed for cytokine production using the following ELISA kits: Human IL‐2 ELISA MAX Deluxe, Human IFN‐gamma ELISA MAX Standard, Human IL‐17A ELISA MAX Deluxe Set (Biolegend, CA), Human IL‐4 ELISA Set (Becton Dickinson, Mountainview, CA), and Human IL‐10 Duoset (R&D Systems).

## Results

### Increased expression of Tim‐1 in PCNSL

**Figure 1:**
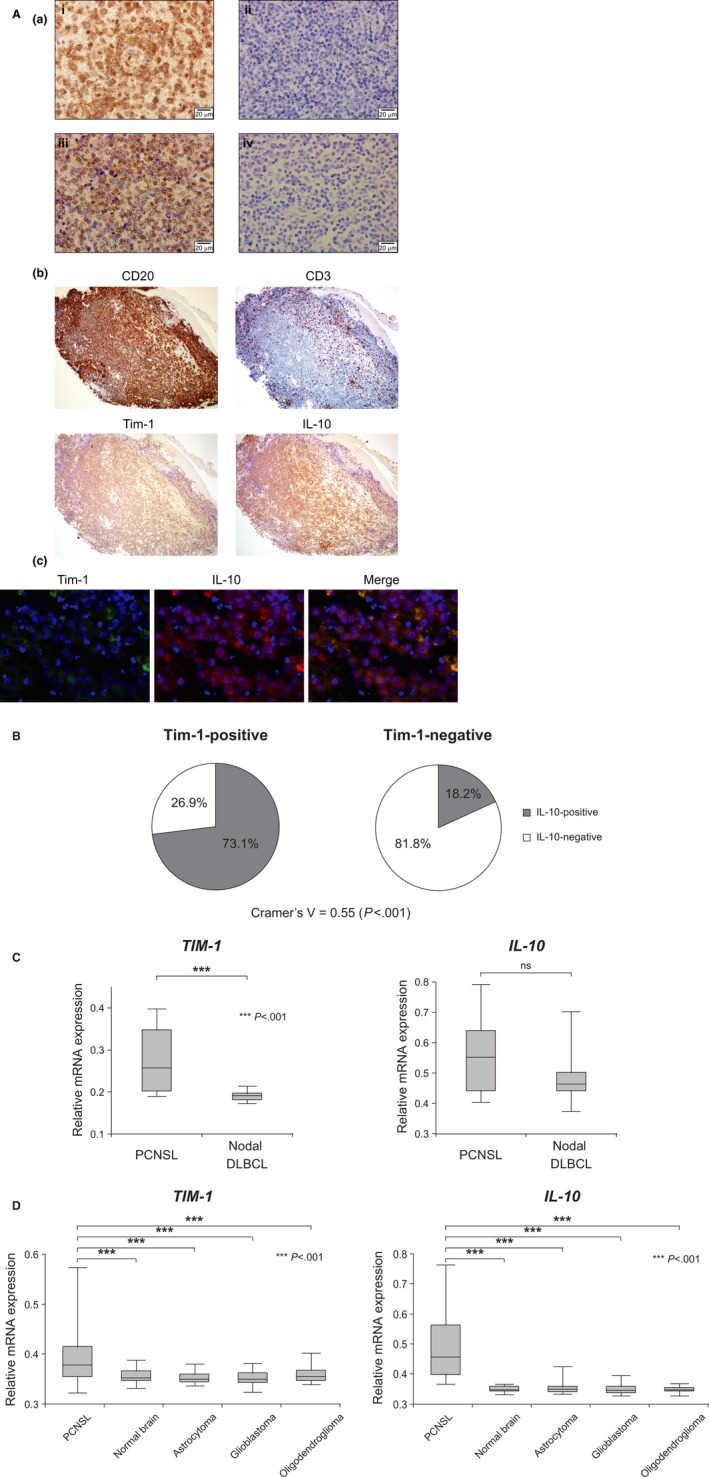
Increased expression of Tim‐1 in PCNSL. (A) (a) Representative immunohistochemical staining patterns of the (i) Tim‐1‐positive and (ii) ‐negative samples, and (iii) IL‐10‐positive and (iv) ‐negative samples (original magnification, ×600). (b) Representative results of the immunohistochemical staining of the serial sections of PCNSL samples (original magnification, ×100). (c) the fluorescent double staining for Tim‐1 and IL‐10 of PCNSL. (B) Pie charts showing IL‐10‐positive rates in the Tim‐1‐positive (left, *n* = 26) and ‐negative (right, *n* = 22) samples. (C) Comparison of the gene expression levels of *TIM‐1* and *IL‐10* between PCNSL (*n* = 9) and nodal DLBCL (*n* = 15) samples using microarray datasets available in the NCBI GEO database (accession number: GSE10524). (D) Comparison of the gene expression levels of *TIM‐1* and *IL‐10* between PCNSL (*n* = 34), normal brain (*n* = 23), astrocytoma (*n* = 26), glioblastoma (*n* = 81), and oligodendroglioma (*n* = 50) samples using microarray datasets available in the NCBI GEO database (accession numbers: GSE4290 and GSE34771). DLBCL, diffuse large B‐cell lymphoma.

We first performed immunohistochemical staining of Tim‐1 and IL‐10 in 48 PCNSL and 89 systemic DLBCL samples (Fig. 1A, a). Tim‐1 and IL‐10 were positive in 17 (19.1%) and 14 (15.1%) of systemic DLBCL. In contrast, they were positive in 26 (54.2%) and 23 (47.9%) of PCNSL (*vs* systemic DLBCL, *P *<* *0.001), and in most positive cases, both Tim‐1 and IL‐10 were stained in more than 80% of the tumor cells. Steroids had been administered before biopsy in 10 PCNSL patients, but they did not seem to affect much on Tim‐1 expression (Cramer's *V* = 0.15). Among systemic DLBCL, Tim‐1 was positive in 9 (26.5%) of 34 germinal center B‐cell like (GCB) and in 8 of 55 non‐GCB DLBCL evaluated by immunohistochemistry based on Hans' algorithm 18, suggesting that Tim‐1 expression was not significantly biased to either cell of origin (*P *=* *0.178). Immunohistochemical staining of the serial sections and fluorescent double staining analysis indicated that Tim‐1 and IL‐10 were coexpressed in tumor B cells (Fig. 1A, b, c), and a moderate correlation was observed between their positivities (Cramer's *V* = 0.55, *P *<* *0.001) (Fig. 1B). In contrast, their correlation was lower in systemic DLBCL (Cramer's *V* = 0.34, *P *<* *0.005). There were five systemic DLBCL samples of the patients who subsequently developed CNS involvement, but all were negative for Tim‐1 while three were positive for IL‐10, and it was suggested that secondary CNS involvement of systemic DLBCL is not associated with Tim‐1 expression of the primary lesions.

We next compared the mRNA expression levels of *TIM‐1* and *IL‐10* in PCNSL and nodal DLBCL by using datasets from the GEO database (accession number: GSE10524 26). The expression of the *TIM‐1* gene was shown to be significantly higher in PCNSL than in nodal DLBCL (*P *<* *0.001), and the expression of *IL‐10* also tended to be higher in PCNSL (Fig. 1C). Additionally, we collected two datasets (accession number: GSE4290 27 and GSE34771 28) measured on the same GPL570 microarray platform, and after normalization using the RMA method, expression levels of *TIM‐1* and *IL‐10* were compared. We found that both genes were expressed significantly higher in PCNSL than in normal brain or other brain tumors (*P *<* *0.001, Fig. 1D). These results suggest that Tim‐1 is expressed in PCNSL with high specificity at both mRNA and protein levels, and it tends to positively correlate with IL‐10 expression.

### Tim‐1 enhances IL‐10 expression in PCNSL

**Figure 2:**
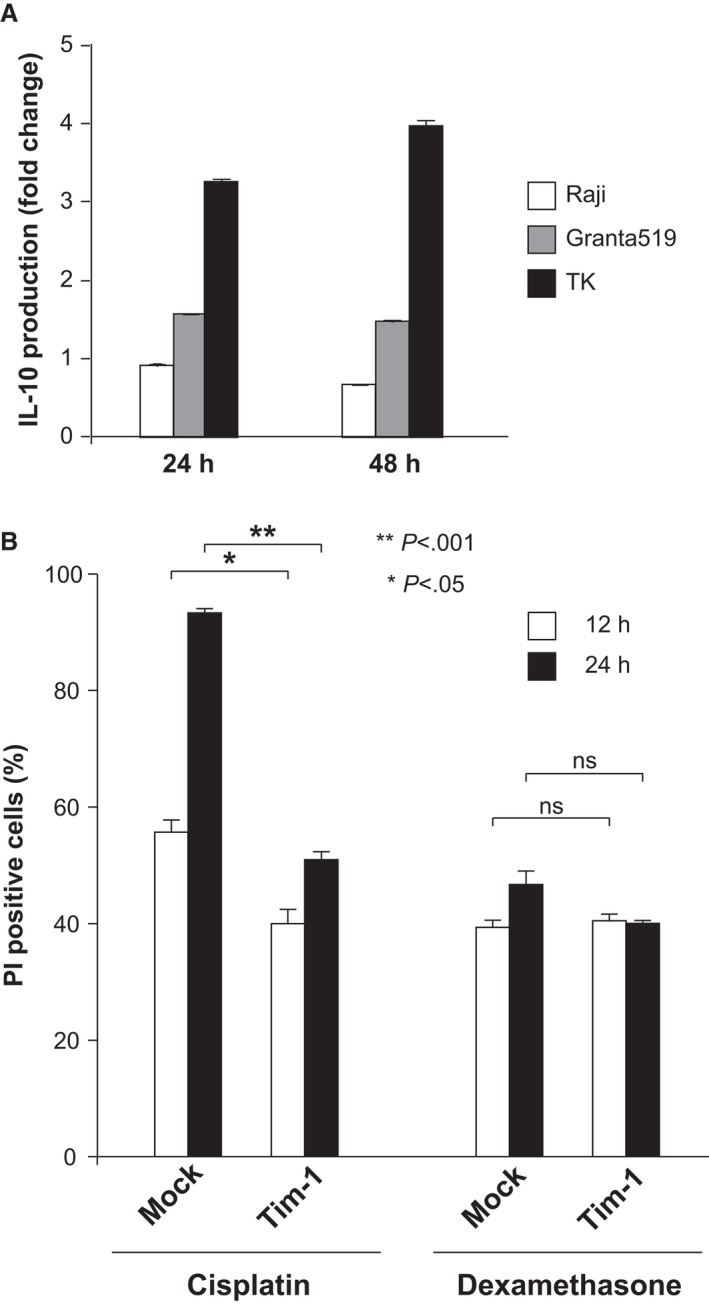
The functional role of Tim‐1 played in PCNSL cells. (A) Comparison of fold changes in IL‐10 production of Raji, Granta519, and TK cells introduced with *TIM‐1*‐expressing and mock vectors. (B) Cell death rates of TK cells introduced with *TIM‐1‐*expressing or mock vectors, which were evaluated by propidium iodide (PI) staining after culturing with cisplatin or dexamethasone for indicated periods.

Next, we intended to clarify the biological relationship between Tim‐1 and IL‐10 expression. In a PCNSL‐derived cell line, TK, endogenous Tim‐1 protein is expressed at a low level. We lentivirally introduced *TIM‐1‐*expressing or mock vector into TK cells, and compared their IL‐10 production by ELISA. Notably, the enhanced expression of Tim‐1 resulted in increased IL‐10 production in TK cells but not in other B‐cell lymphoma lines, Raji and Granta 519 (Fig. 2A), suggesting that Tim‐1 augments IL‐10 expression in a PCNSL‐specific cell condition.

To examine whether Tim‐1 has any role in the survival of PCNSL cells, we cultured *TIM‐1* and mock‐introduced TK cells with cisplatin or dexamethasone. Although the presence of Tim‐1 did not obviously alter cell susceptibility to dexamethasone, it decreased the rate of cell death caused by cisplatin (Fig. 2B), suggesting that Tim‐1 may also confer chemoresistance on PCNSL cells.

### Soluble Tim‐1 in the CSF of PCNSL patients

**Figure 3:**
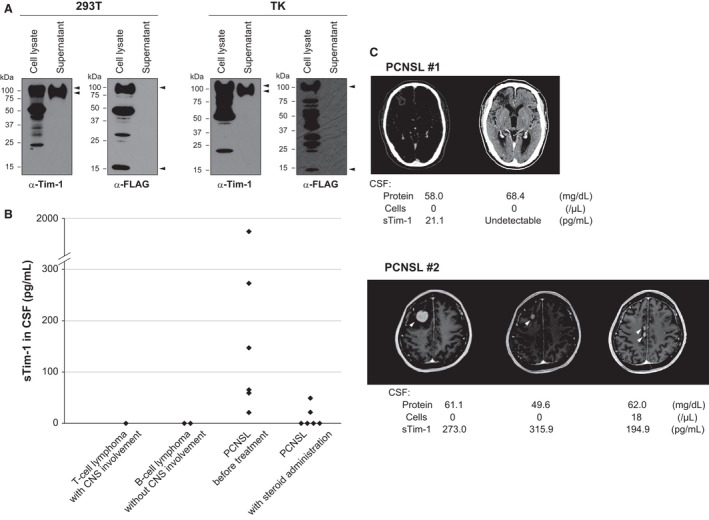
Shedding of Tim‐1 ectodomain and detection of soluble Tim‐1 in the cerebrospinal fluid (CSF) of PCNSL patients. (A) Immunoblot analysis of 293T and TK cells transfected with *TIM‐1* expression vector. The cells and supernatants were separately analyzed for Tim‐1 protein using anti‐Tim‐1 and anti‐FLAG antibodies, which recognize the extracellular domain and FLAG on the C‐terminus, respectively. Arrowheads indicate different forms of Tim‐1 protein: full‐length form (cell lysate), soluble form without C‐terminus (supernatant), and remnant protein (cell lysate). (B) Soluble Tim‐1 levels in the CSF of lymphoma patients analyzed by ELISA. (C) Clinical course of the two representative PCNSL patients. *PCNSL #1*: Tim‐1 in CSF became undetectable after successful treatment of whole‐brain irradiation. *PCNSL #2*: Tim‐1 level remained high after chemotherapy while radiological examination of the tumor suggested a good response to the treatment. The patient subsequently relapsed with multiple brain lesions and CSF dissemination.

As Tim‐1 is expressed in tubular epithelial cells following kidney injury 13 and its soluble form is reported to be released into the urine 14, 15, 16, we examined whether the soluble form of Tim‐1 is also released from PCNSL cells. We transfected *TIM‐1* expression vector into 293T and TK cells, and their supernatants were examined for Tim‐1 protein by immunoblotting (Fig. 3A). Tim‐1 was detected in each supernatant by anti‐Tim‐1 antibody, which reacts with the extracellular domain of the protein, and it was slightly smaller in size than those observed in the cell lysate. On the other hand, Tim‐1 protein was not detected in the supernatant when anti‐FLAG antibody was used, which reacts with the FLAG epitope on the C‐terminus, suggesting that the soluble form of this protein lacks the C‐terminus. Instead, a small remnant protein was detected in the cell lysate by this antibody. These results suggest that, as is reported in tubular epithelial cells, Tim‐1 is cleaved near the C‐terminus and its extracellular domain is released from these cells.

These results led us to examine whether the soluble form of Tim‐1 is also released from PCNSL cells in vivo, and we examined Tim‐1 protein in the CSF of lymphoma patients by ELISA (Fig. 3B). Among 12 CSF samples obtained from PCNSL patients before treatment, Tim‐1 was detected in eight, and the rest four samples were of patients who had been administered systemic steroids before sampling. On the other hand, Tim‐1 was not detected in the CSF of a T‐cell lymphoma patient with secondary CNS involvement, or systemic DLBCL patients without CNS involvement. Soluble Tim‐1 became undetectable in the patients who had achieved complete remission, but it remained positive in the patients with refractory disease (Fig. 3C). In PCNSL patient #2, radiological examination of the tumor suggested a good response to chemotherapy, although Tim‐1 level in the CSF remained high. After 2 months, the patient relapsed with numerous small brain lesions and CSF dissemination. These results suggest that soluble Tim‐1 in the CSF can also reflect the presence of radiologically undetectable tumor cells, and it is expected to serve as a useful biomarker for PCNSL.

### Role of Tim‐1 in the tumor microenvironment

**Figure 4:**
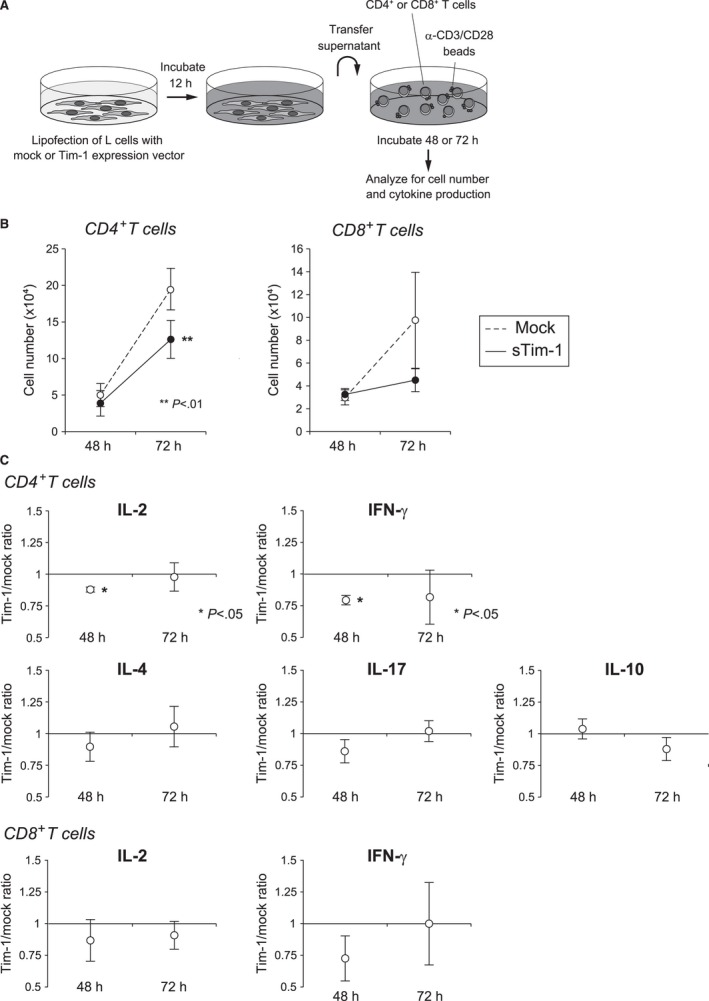
Immunomodulatory effect of soluble Tim‐1. (A) Schematic diagram of the experiment. CD4^+^ and CD8^+^ T cells of the healthy donors were stimulated with anti‐CD3/CD28 beads with or without soluble Tim‐1 produced by L cells, and analyzed for cell proliferation and cytokine production. Soluble form of Tim‐1 was detected in the supernatant of L cells transfected with *TIM‐1* expression vector but not with mock vector (data not shown). (B) Effect of soluble Tim‐1 on T‐cell number. (C) Effect of soluble Tim‐1 on cytokine production of CD4^+^ and CD8^+^ T cells.

To clarify whether soluble Tim‐1 has any role in the tumor microenvironment, we examined its effect on T‐cell function. We collected peripheral CD4^+^ and CD8^+^ T cells from healthy donors, and stimulated them with anti‐CD3/CD28 beads in vitro in the presence or absence of soluble Tim‐1 produced by L cells (Fig. 4A). We found that the proliferation of CD4^+^ T cells was suppressed by soluble Tim‐1 (*P *<* *0.01), and proliferation of CD8^+^ T cells also tended to be inhibited (Fig. 4B). Additionally, IL‐2 and IFN‐*γ* production of CD4^+^ T cells after 48 h of culture was suppressed in the presence of soluble Tim‐1, whereas the production of other cytokines was less affected (Fig. 4C). According to these results, it is suggested that the soluble form of Tim‐1 released from PCNSL cells has some immunomodulatory functions, and it may have a potential role in creating a microenvironment favorable for tumor cells.

## Discussion

In this study, we have reported that Tim‐1, a molecule known as a specific marker for regulatory B cells in mice, is characteristically expressed in PCNSL. About 90–95% of PCNSLs demonstrate DLBCL histology, whereas they exhibit some distinct clinical features from systemic DLBCL, such as site‐confined progression and high responsiveness to methotrexate therapy 29. Histological and molecular studies of PCNSL to date have revealed the frequent expression of MUM‐1 and Bcl‐6 30, 31, 32, high proliferative activity 33, recurrent *BCL6* translocation, and deletion of 6p21.3 34. NF‐*κ*B pathway is generally activated in PCNSL, and gene alterations that lead to its deregulation, such as activating mutations of *CARD11* and *MYD88*, are often involved 35, 36, 37, 38.

Four functional *TIM* genes in mice and three in humans (*TIM‐1*,* TIM‐3*, and *TIM‐4*) have been identified to date, and these *TIM* family members have multiple roles in regulating immune response. Tim‐1 functions as a costimulatory molecule following T‐cell receptor (TCR) signaling, particularly after Th2 polarization 7, 8, 9. Additionally, Tim‐1 has been reported to be a major ligand for endothelial P‐selectin, and have a function to regulate T‐cell trafficking in inflamed tissues 39, 40. Tim‐1 is also expressed in other immune cells such as dendritic cells and mast cells, and plays different roles in each cell type 41, 42, 43, 44.

As for B cells, Tim‐1 was reported to be expressed on a population that has immunosuppressive function, called regulatory B cells, in mice 5. Tim‐1‐positive B cells were shown to be highly enriched for IL‐10 and IL‐4 expression, promote Th2 responses, and be able to transfer allograft tolerance directly. These immunosuppressive B cells have been indicated to participate in the alleviation of brain inflammation in mouse models of multiple sclerosis (EAE) and cerebral infarction 2, 3, 4. Little information is available on the function of B cells in the human brain, but there is a report of nonvasculitic autoimmune meningoencephalitis that developed after rituximab therapy against rheumatoid arthritis 45. Brain biopsy showed a lack of B cells in the brain tissue, and the authors hypothesize that the depletion of regulatory B cells potentially triggered the autoimmune reaction in the brain.

We found high expression of Tim‐1 in PCNSL by histological analysis of the clinical samples. Gene expression analysis using the GEO database also found high Tim‐1 expression in PCNSL compared to other brain tumors, although we were not able to compare with inflammatory brain disorders. Tim‐1 tended to be expressed concurrently with IL‐10, and it was suggested to enhance IL‐10 production in PCNSL cells. Such association between Tim‐1 and IL‐10 was not observed in other B‐cell lines, and is considered to be specific to PCNSL cells. Mouse regulatory B cells with defective Tim‐1 were reported to exhibit low IL‐10 expression 17, suggesting that Tim‐1 is necessary for IL‐10 production in regulatory B cells. The relationship between Tim‐1 and IL‐10 in PCNSL suggests biological similarity of PCNSL to regulatory B cells.

We also found that the extracellular domain of Tim‐1 is released from PCNSL cells, and soluble Tim‐1 is detected in the CSF of PCNSL according to disease activity. An increased IL‐10/IL‐6 ratio in the CSF is known to be useful for the diagnosis of PCNSL, but it is often difficult to measure accurately in clinical practice because of its instability. The half‐life of Tim‐1 is longer than that of IL‐10 (about 6 h *vs* 1–2.6 h) 46, 47, 48, and it can be a more clinically applicable biomarker, although further examination is required. In contrast, serum Tim‐1 levels varied among healthy controls irrespective of renal function in our preliminary analysis, and it did not seem to be useful as a biomarker.

In summary, we found that Tim‐1 is characteristically expressed in PCNSL. In addition to its potential usefulness as a clinical biomarker, Tim‐1 is expected to be a key molecule for the better understanding and improved clinical management of PCNSL.

## Conflicts of Interest

The authors have no financial conflicts of interest to disclose with regard to this report.
